# Large Mesenchymal Hepatic Hamartoma in Pediatric Age: A Case Report

**DOI:** 10.1155/cris/1929050

**Published:** 2025-04-30

**Authors:** Alhasan H. Alhebshi, Ammar Kabbarah, Murad Aljiffry

**Affiliations:** ^1^Department of surgery, International Medical Center, Jeddah, Saudi Arabia; ^2^Department of Surgery, King Abdulaziz University, Jeddah, Saudi Arabia; ^3^Department of Surgery, Faculty of Medicine, King Abdulaziz University, Jeddah, Saudi Arabia

**Keywords:** hepatic mesenchymal cyst, liver cyst, rare liver tumor

## Abstract

Benign liver tumors are infrequently observed in the pediatric age group, with an incidence reported at 0.7 per million population annually. Among these tumors, mesenchymal hamartoma constitutes 18%–29%. Imaging studies commonly reveal a well-marginated, solitary mass, often measuring up to 30 cm. The mass, primarily located in the right liver lobe (75% of cases), may exhibit a pedunculated structure. We present a case of a 1-year-and-9-month-old boy diagnosed with hepatic mesenchymal hamartoma. A contrast-enhanced computed tomography of the abdomen and magnetic resonance imaging (MRI) were performed and demonstrated a large multiloculated septated liver lesion measuring approximately 13.6 × 17.7 cm, demonstrating multiple partially thickened internal septations. The procedure was done for the patient in the form of an extended right hepatectomy with segment 4A and cholecystectomy.

## 1. Introduction

Benign liver tumors are infrequently observed in the pediatric age group, with an incidence reported at 0.7 per million population annually. Among these tumors, mesenchymal hamartoma constitutes 18%–29% [1].

Common clinical presentations include progressive abdominal distention, accompanied by a palpable, asymptomatic mass in the right upper quadrant [2].

Imaging studies commonly reveal a well-marginated, solitary mass, often measuring up to 30 cm. The mass, primarily located in the right liver lobe (75% of cases), may exhibit a pedunculated structure. Calcifications and hemorrhage are rare, and an unenhanced CT scan shows a heterogeneous appearance. The mesenchymal component enhances after contrast administration [3], and the imaging appearance varies based on the prevalence of stromal or cystic elements. MR imaging appearances depend on the cystic versus stromal composition, as well as the protein content of the fluid [2, 4, 5].

Treatment typically involves surgical resection, revealing a mass composed of loose edematous tissue, blood vessels, hepatocytes, abnormal bile ducts, and immature mesenchyme. Differential diagnoses encompass various intraperitoneal cystic masses, such as hydatid cysts, mesenteric cysts, gastrointestinal duplication cysts, and cystic teratomas of the mesentery [6–10].

## 2. Case Report

A 1-year-and-9-month-old boy with no medically relevant personal or family history. Parents noticed abdominal distention with discomfort and mass effect for 4 months. No other complaints. Physical examination revealed a firm mass, nontender is occupying most of the abdominal cavity. Laboratory studies showed normal LFT except for increasing ALP 168 (46–116) IU and GGT 133 IU (<73) with normal complete blood count and normal renal function. The hydatid cyst blood investigations were negative. Alpha -fetoprotein blood work was normal.

An abdominal ultrasound (US) was performed and demonstrated a large abdomopelvic multiloculated and multiseptated mass lesion measuring about 10.5 × 17.5 × 18 cm. The liver and the gallbladder could not be visualized.

**Figure 1:**
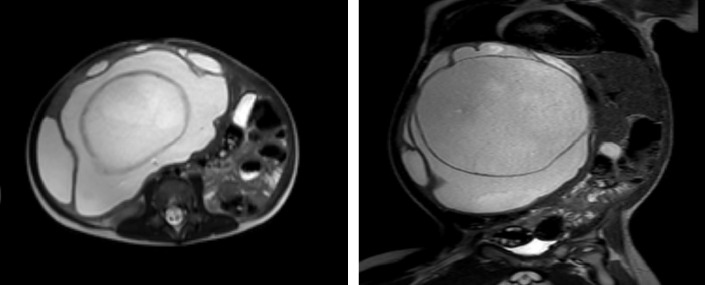
MRI abdomen showing a large 13.6 × 17.7 cm.

For further characterization. A contrast-enhanced computed tomography of the abdomen and magnetic resonance imaging (MRI) were performed, which revealed a large multiloculated and multiseptated liver lesion measuring approximately 13.6 × 17.7 cm demonstrating multiple partially thickened internal septations. It demonstrated bright T2 signal intensity, low T1 signal intensity without evidence of diffusion restriction. (Figure 1)

The lesion showed minimal enhancement of the internal septations no evidence of a definite solid component. There was minimal intrahepatic biliary ductal dilatation within the right hepatic lobe. There was no evidence of left intrahepatic biliary ductal dilatation. The rest of the liver parenchyma was homogeneous with no focal lesions.

**Figure 2:**
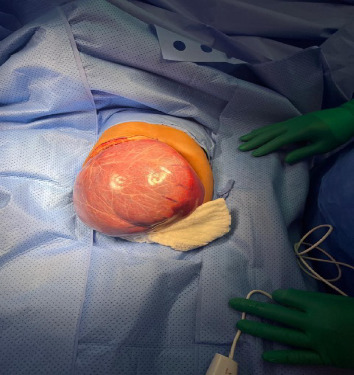
Large solid cystic lesion.

**Figure 3:**
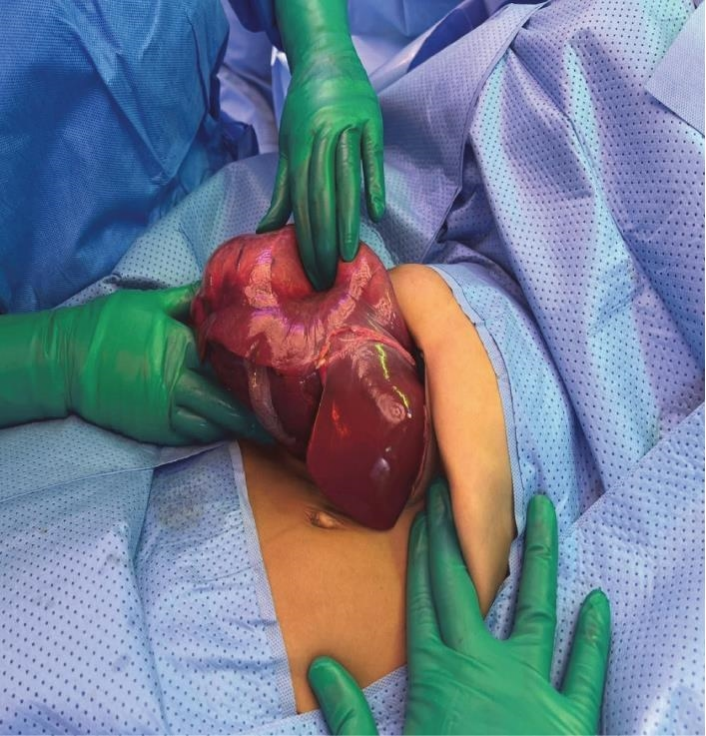
Lesion occupying all right liver lobe extending into segment 4A.

He underwent surgical exploration with a mini laparotomy incision that was extended to Fish hook (L shaped incision) due to difficulty in exposure as the hepatic cyst was occupying most of the abdomen, exploration of the abdomen did not reveal any disease other than large solid cystic lesion (Figure 2), occupying all right liver lobe extending into segment 4A. After liver mobilization and identification of cyst margins (Figure 3), a small puncture was made over the most prominent area of the cyst with purse-string sutures made around the puncture side prior to incision.

**Figure 4:**
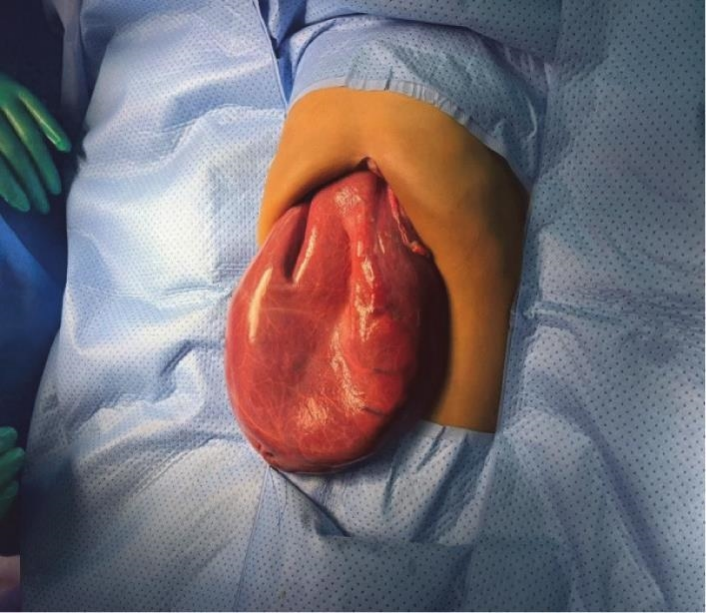
Deflated hepatic cyst.

**Figure 5:**
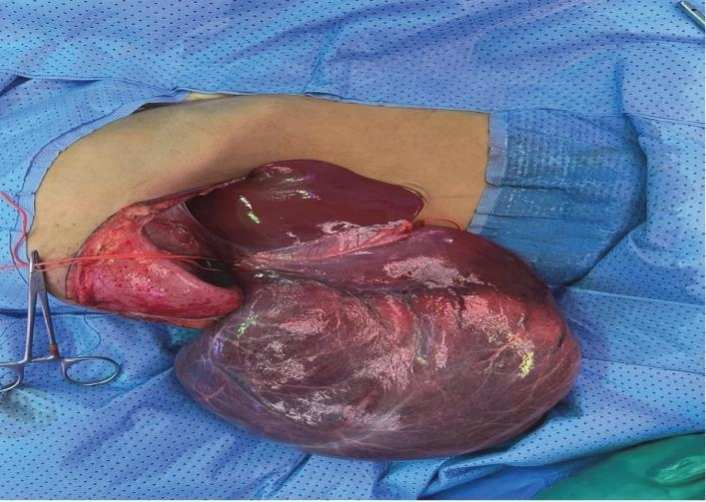
After mobilization of liver.

The contents of the cyst were carefully aspirated, with approximately 1.5 L of fluid removed to achieve deflation. (Figures 4 and 5).

**Figure 6:**
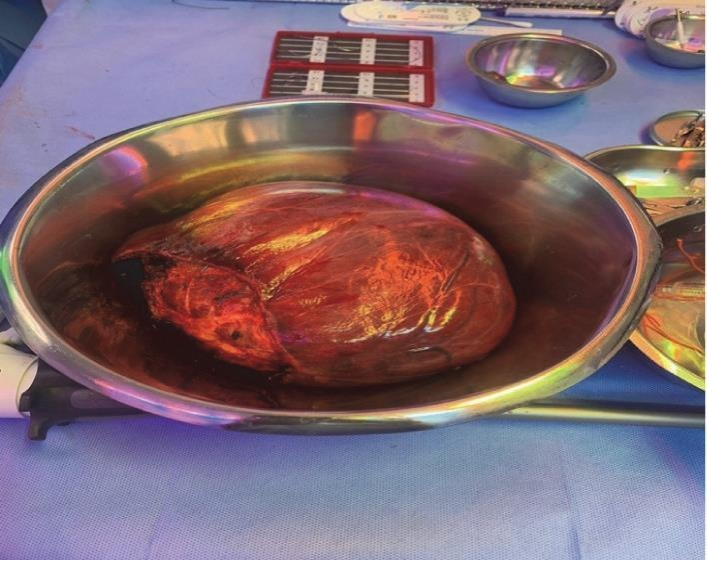
Removed liver segment with hepatic cyst.

Then resection was done in the form of extended right hepatectomy with segment 4A and cholecystectomy with no complications intraoperatively. The removed segment (Figure 6) and fluid aspirated collectively weighed 2318 g.

The patient had an uneventful recovery and was discharged after 6 days in good condition. The patient was seen in the clinic 1 week after discharge with no active complaint and tolerating oral feeds and a well-healed wound.

Histopathological examination showed hepatic mesenchymal hamartoma (17 cm), 0.1 cm from the resected margin, and gallbladder with no pathologic changes.

## 3. Discussion

Liver mesenchymal hamartoma (LMH) is a rare, benign liver lesion predominantly observed in pediatric patients. It is the second most common liver tumor in children, following hepatoblastoma, and accounts for approximately 8% of all pediatric tumors. LMH generally presents in children under 5 years of age, including cases identified prenatally. However, more than two-thirds of cases occur in children younger than 2 years [2]. In our case, we present a male patient with an age of 1 year and 9 months in the typical age of presentation.

Our case presented to us complaining of Abdominal distention and abdominal mass. In the literature,

mesenchymal liver hamartoma has common clinical presentations, which include progressive abdominal

distention, accompanied by a palpable, asymptomatic mass in the right upper quadrant; furthermore, it has a clinical association with conditions such as polycystic kidney disease, congenital hepatic fibrosis, and biliary hamartoma. LMH can be associated with other pathologies and has been linked to chromosomal abnormalities, particularly involving chromosome 19 [2, 5].

Working diagnosis for mesenchymal hepatic hamartoma includes hydatid cyst of the liver, hepatoblastoma, fibrolamellar hepatocellular carcinoma, lymphangioma, cystic teratoma, and hemangioendothelioma (Table 1), and many more, and it is differentiated based on imaging studies, such as US, CT scan, and MRI. Differentiation can be done based on blood works, such as alpha fetoprotein in hepatoblastoma and positive hydatid cyst blood workup [12]. In our case, Alpha fetoprotein was negative and hydatid cyst blood workup, in terms of indirect hemagglutination test and ELISA was negative.

In our case, we initially ordered an US abdomen for the patient to investigate the reason for the abdominal distention and to characterize the etiology, then a contrast enhanced computed tomography of the abdomen and MRI was performed, revealing a large multiloculated multi septated liver lesion measuring approximately 13.6 × 17.7 cm demonstrating multiple partially thickened internal septation, the lesion is demonstrating minimal enhancement of the internal septations. There was no evidence of a definite solid component.

While the cystic component usually prevails in mesenchymal hamartoma (Table 2), considerations for solid appearances in imaging are essential. This underscores the importance of distinguishing it from hepatoblastoma, infantile hemangioendothelioma, hepatocellular carcinoma fibrolamellar type, and metastatic lesions, as their presentations and treatments differ [12].

LMH typically lacks malignant potential, and it is considered a developmental anomaly rather than a

neoplasm, but it often necessitates total surgical resection due to its possible malignant transformation. With an overall favorable prognosis and rare reported cases of recurrence post-surgery. Because of the possibility of a malignant mesenchymoma arising from mesenchymal hamartoma, recommendations the optimal management of LMH remain a subject of debate. Although spontaneous regression has been reported, surgical resection is generally considered the standard treatment, aiming to preserve sufficient liver tissue to maintain normal function [13]. Mortality is primarily associated with surgical complications [5]. Most of the mesenchymal liver hamartomas occur in the right lobe, with a percentage of 75%, the remaining percentage occurs in the left lobe or involves both lobes [14].

In our case, the liver cyst was occupying all right liver lobe, extending into segment 4A, which accounts for part of 25% of mesenchymal liver hamartomas in the literature. Surgery performed for our case in the form of an extended right hepatectomy with segment 4A and cholecystectomy, with clear margins proven by histopathology findings.

## 4. Conclusion

Hepatic mesenchymal hamartoma is a rare, benign hepatic tumor with a different appearance on imaging. The variability extends from a multilocular cystic mass with a solid septum, to multiple small cysts in as solid mass. The definite treatment is surgical excision with a high cure rate.

## Figures and Tables

**Table 1:** Differential diagnosis table for mesenchymal hamartoma of the liver [11].

Diagnosis	Ultrasonography	Computed tomography	MR imaging
Mesenchymal hamartoma of the liver	Large, intraperitoneal mass. Solid component and multiple cysts of variable sizes. Solid septa. Calcifications and hemorrhage are rare but may occur.	Heterogeneous appearance. Central water attenuation areas. Peripheral thick, heterogeneous area, which enhances after contrast injection. Calcifications and hemorrhage.	Solid areas are hypointense both on T1 and T2. Cystic areas have water signal intensity on T2 and variable signal on T1. Enhancement is mild and limited to the septa and stromal components.

Hydatid cyst of the liver	Variable findings according to the evolution phase: 1-Symple cysts. 2-Cysts with internal membranes. 3-Cysts with internal daughter cysts and debris. 4-Densely calcified masses	Well-defined wall. Homogeneous water attenuation cystic components or heterogeneous cystic components with internal membranes or multiple daughter cysts. Peripheral calcifications.	The pericyst is seen as a hypointense rim on both T1 and T2. The hydatid matrix appears hypointense on T1 and markedly hyperintense on T2. Daughter cysts are hypointense on both T1 and T2.

Enteric duplication cyst	Unilocular cysts. Thick double-layered wall. Intracystic hemorrhage may occur.	Water attenuation masses with tick wall. Enhancement of the wall after contrast administration.	Water signal intensity on T2. Thick wall, which enhances after gadolinium administration.

Lymphangioma	Large cystic masses. Thin walled. Multilocular. Anechoic or containing internal echoes and debris.	Variable density. Water attenuation lesions (serous content) to fat attenuation lesions (chylous content).	Cyst content intensity similar to water on T1 and T2-weighted images.

Cystic teratoma	Heterogeneous mass. Pure cystic components. Calcifications.	Heterogeneity, with areas of water and fat attenuation. Calcifications.	Signal intensity characteristics of fat (hyperintense on T1) and water (hypointense on T1 and hyperintense on T2).

Hepatoblastoma	Well circumscribed solid hyperechoic mass.	Slightly hypoattenuating. Calcifications and hemorrhage are common. Enhancement after contrast administration, but less than normal liver.	Slightly hypointense on T1 and hyperintense on T2. Fibrotic septa are hypointense on both T1 and T2-weighted images.

Hemangio-endothelioma	Hypoechoic or of mixed echogenicity. Calcification, hemorrhage, necrosis, and fibrosis in larger lesions. Color doppler shows enlarged vessels.	Well-defined hypoattenuating mass. Calcification and hemorrhage. After contrast administration the enhancement pattern is similar to that of the hemangiomas.	Hyperintense on T2-weighted images. After gadolinium administration enhancing pattern is similar to that seen on CT.

Fibrolamellar HCC	Heterogeneous, hypoechoic or isoechoic. The central scar is hyperechoic, with or without calcifications.	Hypoattenuating mass. Contrast enhancement in arterial phase. Variable wash-out in portal and venous phases. The central scar is hypoattenuating and it may have some enhancement on delayed phase.	Typically hypointense on T1 and hyperintense on T2. Fibrous central scar and septa hypointense on both T1 and T2-weighted images.

Metastases	Multiple solid lesions or diffuse infiltration of the liver. Cystic lesions or solitary solid masses are rare.	Often hypoattenuating masses. Variable appearance and patterns of contrast enhancement.	Most metastases are hypo to isointense on T1 and iso to hyperintense on T2. Variable enhancement.

**Table 2:** Summary table for mesenchymal hamartoma of the liver [11].

Etiology	Not completely understood, probably an abnormality of the ductal plate development.

Incidence	Rare. Less than 0.2 cases per million population per year.

Gender ratio	Male predominance with a 3:2 male:female ratio.

Age predilection	Average age 15 months. 95% of the lesions occur in children younger than 5 years old.

Risk factors	Polycystic kidney disease, congenital hepatic fibrosis and biliary hamartomas are associated anomalies.

Treatment	Surgical excision.

Prognosis	Excellent. Mortality is related to surgical complications.

Findings on imaging	Large, solitary, and cystic mass. Ranges from a solid mass with multiple small cysts, to a multilocular cystic mass with solid septa. After contrast administration the solid component enhances. Calcifications and hemorrhage are rare, but may occur.

Findings on pathology	Edematous tissue, blood vessels, small groups of hepatocytes, abnormal bile ducts, and immature mesenchyme in variable proportions. Areas of degeneration and fluid accumulation.

## Data Availability

The authors confirm that the data supporting the findings of this study are available within the article.
